# Development and validation of a video on bed baths*

**DOI:** 10.1590/1518-8345.3655.3329

**Published:** 2020-08-12

**Authors:** Juliana de Lima Lopes, Rui Carlos Negrão Baptista, Tânia Arena Moreira Domingues, Rosali Isabel Barduchi Ohl, Alba Lucia Bottura Leite de Barros

**Affiliations:** 1 Universidade Federal de São Paulo, Escola Paulista de Enfermagem, São Paulo, SP, Brazil.; 2 Escola de Enfermagem de Coimbra, Coimbra, Portugal.

**Keywords:** Baths, Validation Studies, Video-audio Media, Nursing, Education, Nursing, Instructional Films and Videos, Banhos, Estudos de Validação, Mídia Audiovisual, Enfermagem, Educação em Enfermagem, Filmes e Vídeos Educativos, Baños, Estudios de Validación, Medios Audiovisuales, Enfermería, Educación en Enfermería, Películas y Videos Educativos

## Abstract

**Objective:**

develop and validate a video on bed bathing directed to nursing professionals and students.

**Method:**

the video was based on the literature and presents the definition of bed bath, indications for its performance, steps to perform it, and potential complications. Nursing professors and nurses validated it. They assessed the pertinence of content, clarity, and language of the video scenes. The Delphi Technique was used in this phase. After recording, three nursing professors, along with undergraduate nursing students from a public university, assessed the educational video. The professors first watched the video and suggested changes, and then the students watched the video after the changes were implemented.

**Results:**

six rounds were needed for experts to validate the video script using the Delphi Technique. After the video recording, undergraduate students considered the video of easy understanding.

**Conclusion:**

the video script was composed of four topics and was validated by experts after six rounds. The video was assessed by the professors and nursing undergraduate students, who considered the topics and the video as a whole as apprehensible. This study is expected to contribute to professional training and improvement of the knowledge and skills of nursing students.

## Abstract (pt)

**Objetivo:**

elaborar e validar um vídeo sobre a técnica do banho no leito direcionado aos profissionais e estudantes de enfermagem.

**Método:**

o vídeo foi baseado na literatura e apresenta a definição de banho no leito, indicações, passos para sua realização e possíveis complicações. A validação foi conduzida por docentes de enfermagem e enfermeiros, que avaliaram a relevância do conteúdo, clareza e linguagem utilizada no vídeo. A Técnica de Delphi foi usada nesta fase. Depois da gravação, o vídeo educacional foi avaliado por três docentes de enfermagem e estudantes do Curso de Graduação em Enfermagem de uma universidade pública. Primeiramente os docentes assistiram ao vídeo e sugeriram alterações e, posteriormente, após os ajustes solicitados, os alunos assistiram ao vídeo.

**Resultados:**

seis rodadas foram necessárias para os especialistas validarem o roteiro do vídeo usando a Técnica de Delphi. Depois de assistir ao vídeo, os alunos de graduação o consideraram compreensível.

**Conclusão:**

o roteiro do vídeo, composto por quatro tópicos, foi validado por especialistas depois de seis rodadas. O vídeo foi avaliado por docentes e alunos de graduação, que consideraram os tópicos, e o vídeo como um todo, compreensíveis. Espera-se que este vídeo possa contribuir para a qualificação profissional e aprimoramento do conhecimento e habilidades de estudantes de enfermagem.

## Abstract (es)

**Objetivo:**

elaborar y validar un video sobre la técnica del baño en la cama dirigido a los profesionales y estudiantes de enfermería.

**Método:**

el video fue basado en la literatura y presenta la definición de baño en la cama, indicaciones, pasos para su realización y posibles complicaciones. La validez fue realizada por docentes de enfermería y enfermeros, los que evaluaron la relevancia de contenido, clareza y lenguaje, utilizados en el video. La Técnica de Delphi fue utilizada en esta fase. Después de la grabación, el video educacional fue evaluado por tres docentes de enfermería y por estudiantes del curso de graduación en enfermería de una universidad pública. Primero, los docentes presenciaron el video y sugirieron hacer alteraciones; posteriormente, después de los ajustes solicitados los estudiantes presenciaron el video.

**Resultados:**

seis rondas fueron necesarias para que los especialistas validasen el guión del video, usando la Técnica de Delphi. Después de ver el video, los estudiantes de graduación lo consideraron comprensible.

**Conclusión:**

el guión del video, compuesto por cuatro tópicos, fue validado por especialistas después de seis rondas de análisis. El video fue evaluado por docentes y estudiantes de graduación, los que consideraron los tópicos y el video como siendo un todo comprensible. Se espera que este video pueda contribuir para la calificación profesional y el perfeccionamiento del conocimiento y de habilidades de los estudiantes de enfermería.

## Introduction

Bed bathing is one of the procedures most frequently performed in clinical practice, but because its execution presents various steps, students often face difficulties performing it^(^ 1 ^)^. Therefore, teaching tools that aid in the execution of this procedure are extremely important. Universities currently seek other tools to use in the teaching-learning process with students, since teaching based only on theoretical classes does not facilitate the retention of knowledge^(^ 2 ^)^. Thus, various tools have been used, and videos are among them.

A video can be an effective tool to be used during classes because it has various advantages, such as the fact it is a practical method, with a good cost-benefit profile, and low investment when compared to the large number of people it reaches. It also facilitates understanding of information as people are allowed to watch the scenes multiple times, as many times as necessary, and is well-accepted in the educational field^(^ 3 ^-^ 4 ^)^. This tool can be used in undergraduate teaching, as well as to improve the knowledge of patients and family members who are supposed to perform a given procedure^(^ 5 ^-^ 8 ^)^. Video can be used in various teaching settings, such as classrooms, simulation centers, distance learning, and can aid students in acquiring new skills and improve their learning process.

Compared with written information, videos are more accessible in terms of language and communication and can be more cost-effective^(^ 9 ^)^. Video content allows for the demonstrations of clinical skills that undergraduate students are yet to experience, thus providing them an opportunity to enhance visual learning.

There are various methods to develop a video but in general three steps are implemented: pre-production (development of a script, considering the target population and filming strategies), production (video recording, considering appropriate lighting and positioning of cameras and microphone) and post-production (video editing and inclusion of audio)^(^ 10 ^)^. Another important step is assessing the video, which can be performed by experts and by the population who are supposed to use the video^(^ 3 ^,^ 11 ^)^. In this context, studies developing and validating videos addressing nursing procedures are essential both for teaching and care delivery.

One study, the objective of which was to develop and validate an educational video on the dressing of central venous catheters without cuff, shows that this tool can contribute to nurses’ training and can be used to update human resources, even through distance learning, improving the quality of care delivery^(^ 12 ^)^. Other studies^(^ 8 ^,^ 11 ^,^ 13 ^-^ 14 ^)^ report the efficacy of using videos, such as reducing the need to repeat lectures, and allowing to review and improve teaching methods^(^ 11 ^)^; increase knowledge at the short-term^(^ 8 ^)^; and improve practical and theoretical knowledge and performance in assessment tests^(^ 13 ^-^ 14 ^)^.

From this perspective, nurses can develop and incorporate validated teaching technologies, such as videos, to assist educational practices. However, no studies developing and validating a video on bed bathing were found in the literature. This procedure has several steps and can lead to complications when not performed appropriately. Therefore, a video can promote patient safety, as students are allowed to practice several times while watching the video before actually providing patient care.

In this sense, the following guiding question emerged: Do nurse specialists and undergraduate nursing students consider a literature-based video considered valid?

In order to answer this question, the objective was to develop and validate a video on bed bathing directed to nursing professionals and students.

## Method

This methodological study was conducted from July 2016 to January 2017. The video’s development and validation were performed according to the steps described by other authors: development and validation of the video script, recording, and video assessment^11-12^.

The video script, a storyboard, was based on the literature. Three professors from the Fundamental Nursing Program and four nurses with at least two years of hospital experience validated the video script, all of whom consented to participate and signed free and informed consent forms. Specialists teaching classes on the topic and/or providing care to patients who required bed baths were selected. The specialists were recruited through intentional non-probabilistic sampling on the Lattes Platform. Note that the Delphi Technique allows the number of experts to be directly determined by the phenomenon one intends to study, and there is no consensus in the literature on the ideal number of experts; studies validating video scripts have selected from five to nine specialists^(^ 3 ^,^ 11 ^,^ 15 ^)^.

The nurses assessed the pertinence of content, clarity, and language used by the characters of each of the video scenes^(^ 11 ^)^, as well as the order of dialogues and images. An instrument containing all the topics addressed in the video that included a three-point Likert scale (3 totally appropriate, 2 partially appropriate, and 1 totally inappropriate), used in previous studies, was adopted^(^ 11 ^)^ and sent via e-mail along with the video script to be analyzed. If nurses considered the script to be partially appropriate or totally inappropriate, they were asked to suggest pertinent changes. In order to be considered valid, there should be a 100% agreement regarding the video script among the specialists and achieve a mean score equal to 3 (totally appropriate).

The Delphi Technique was used in this phase of validation. This technique has been used to validate instruments and video scripts^(^ 3 ^,^ 11 ^,^ 16 ^)^. It is a method to collect opinions and criteria from a group of experts on a given topic by successively applying questionnaires, while information from previous phases is used in each phase to reach consensus among the participants^(^ 17 ^)^. Comments from one round are collected, analyzed, and integrated into the next round to collect opinions from the experts. The advantages of using the Delphi method are: condensing different opinions and obtaining consensus among experts; the anonymous nature of the process, which prevents a single strong individual from dominating the process and influencing the group’s opinion; the process can be carried out via e-mail and does not require experts to physically meet, enabling members from different places to participate^(^ 18 ^)^.

The recording of this educational video was based on the validated script. The video was developed together with a team specializing in video development, which recorded the video scenes and the characters’ lines. It was recorded in a Skills and Simulation Center, where a scenario was created with a fictitious infirmary. Those who participated as part of the cast became familiar with the script before recording and editing. The video was composed of images, scenes, and descriptive phrases. The scenes were filmed repeated times by the Skills and Simulation Center at the Federal University of São Paulo until the content of the validated script was complete; ten hours of work were necessary to film all the scenes. The scenes that contained the patient’s phrases were filmed first because the actor (a nurse) performed these scenes first, and then the validated script was followed. The technique of the procedure was simulated by two nurses using a low-detail training manikin. Two Sony Nx5 camcorders were used. A professional with experience in voice-over recorded the narration of the video in a studio with an Audio-technica microphone and Tascam recorder, while the video was edited using Adobe Premiere CC Program. Three days were necessary to complete the editing, in order to include the content of the validated script, totaling ten hours. One re-edit was required at this point.

Three professors from the Fundamental Nursing Program assessed the educational video for content and face validation and undergraduate nursing students from a public university in São Paulo, Brazil assessed it for face validation. Professors first watched the video and suggested changes. After these changes were implemented, the students watched the video. The population was composed of second-year nursing students who agreed to participate in the study. Sophomore students were selected because the theoretical classes addressing the topic of bed bathing are taught during the second year of the program. A five-point Likert scale, developed by the researchers and used in a previous study^(^ 11 ^)^, was used for the students to assess understanding of the educational video as a whole and each of its parts. The minimum score was 1 (I did not understand anything), and the maximum was 5 (I understood it perfectly and have no doubts). In addition to the content, students were asked to assess the video for images and audio, the environment where it was filmed and the characters. The Wilcoxon Test was used to verify agreement among the students’ answers. Binomial distribution was used to calculate a 95% confidence interval for the proportion of maximum scores (equal to 5). The project was submitted to the Institutional Review Board, while data collection was initiated only after receiving approval (No. 1025890).

## Results

The video script was composed of four topics addressing the definition of bed bathing, the indications for this type of bath, steps (orientation provided to the patient, preparation of material, and bath technique), and related complications. After its development, the video script was validated by nurses (both nursing professors and professional nurses); six rounds were necessary to obtain a consensus among the experts. Note there were no specialists losses during the rounds, and no changes were requested regarding the definition of the procedure. Tables 1 and 2 show the mean scores improved throughout the rounds.

**Table 1:** Mean scores of the topic "indication" in the six rounds. São Paulo, SP, Brazil, 2016-2017

	Indication			
	Content	Clarity	Order of dialogue	Language
Round 1	2.95	2.71	3.00	2.86
Round 2	2.79	2.79	3	2.93
Round 3	2.79	2.93	3	3
Round 4	3	3	3	2.86
Round 5	3	3	3	3
Round 6	3	3	3	3

**Table 2:** Mean scores of the topic "procedure" and "complications" in the six rounds. São Paulo, SP, Brazil, 2016-2017

	Procedure				Complications			
	Content	Clarity	Order of dialogue	Language	Content	Clarity	Order of dialogue	Language
Round 1	2.52	2.52	2.67	2.57	2.43	2.71	2.86	2.71
Round 2	2.98	2.97	3	2.94	2.98	2.98	3	3
Round 3	2.99	2.98	3	2.98	3	2.9	3	2.81
Round 4	3	2.95	3	2.94	2.95	3	3	2.95
Round 5	3	2.97	3	2.95	2.93	3	3	3
Round 6	3	3	3	3	3	3	3	3

Regarding the topic “definition”, three rounds were necessary to obtain a consensus among the experts. In round 1, the mean score of the content was 3.0; clarity was 2.71; order of dialogue was 3.00; and language was 2.29. In round 2, the mean score of content was 3.00; clarity was 3.00; and language was 2.57.

The changes suggested by the nurses regarding the content can be viewed on Figure 1.

**Figure 1:** Changes suggested by the nurses. São Paulo, SP, Brazil, 2016-2017

Topic	Round 1	Round 2	Round 3	Round 4	Round 5
Indication	To include indication of bed bathing for patients with self-care disorders	To include examples of changes in self-care.	Text order changed.	No change was proposed.	No change was proposed.
Procedure	To include phrases related to patient safety (raise bed rails, remove the plastic covering venous access after the bath, and concomitantly clean the patient's bed and furniture with 70% alcohol solution).	To include phrase related to inform of the need to practice hygiene of the upper and lower limbs on the opposite side of the professional washing the patient and include a modified technique of the procedure for patients with reduced mobility and/or who are unconscious.	To include the justification of why not to apply comforting massage vigorously on bone prominences and where pressure injuries are starting to appear (stage I) and keep the call-light close to the patient after bathing. The text order was changed regarding the procedure for unconscious patients or patients with reduced mobility.	No change was proposed.	No change was proposed.
Complications	To include other bath-related complications (microbial translocation).	To include interventions to prevent complications such as changing the dressings affixing tubes and probes after bathing.	To include (endotracheal) tube specificities.	To include interventions to prevent complications such as covering the peripheral venous access to prevent infection.	To include information about keeping patients in severe condition under hemodynamic monitoring.

An educational video on bed baths of 21 minutes and 21 seconds duration was developed addressing the following topics: definition (36 seconds), indication (21 seconds), procedure (17 minutes and 24 seconds), and complications (3 minutes).

After the video was developed, three professors from the Fundamental Nursing Program assessed it and proposed small adjustments (include hand-washing after organizing the room, increase the sound of the patient’s voice and that of the professionals in the video, and change the term from ‘pressure ulcer’ to ‘pressure injury). The video was re-edited implement these changes and then the video was presented once more to the nurses; no further changes were suggested at that time.

Afterwards, the video was assessed by the nursing undergraduate students, who scored the level of comprehension for each topic and the video as a whole. All scores were greater than 4 (Table 3) and the percentage of maximum scores was high, ranging from 77% to 95% (Table 4); i.e., the video was well understood by the students.

**Table 3:** Descriptive analysis of the students' assessments and test to verify whether the means of answers were greater than 4. São Paulo, SP, Brazil, 2016-2017

Variable	Mean	SD*	SE^†^	Median	IQ^‡^	Min^§^	Max^‖^	N	p-value (Wilcoxon Test)
Video as a whole	4.75	0.47	0.06	5	0	3	5	64	<0.0001
Definition	4.81	0.43	0.05	5	0	3	5	64	<0.0001
Indication	4.95	0.21	0.03	5	0	4	5	64	<0.0001
Procedure	4.77	0.5	0.06	5	0	3	5	64	<0.0001
Complications	4.78	0.49	0.06	5	0	3	5	64	<0.0001

* SD = Standard deviation;

† SE =Standard error;

‡ IQ = Interquartile;

§ Min = Minimum

‖ M =maximum

**Table 4:** Descriptive analysis of students' assessments and confidence intervals of the proportions of maximum scores. São Paulo, SP, Brazil, 2016-2017

Variable	Frequency of 5's	% of 5's	n	CI* 95%	
				Upper	Lower
Video as a whole	49	77%	64	64%	86%
Definition	53	83%	64	68%	89%
Indication	61	95%	64	87%	99%
Procedure	51	80%	64	68%	89%
Complications	52	81%	64	70%	90%

* CI = Confidence interval

## Discussion

Various steps that can be taken to develop a video, but the most frequently used are: planning or pre-production, filming or production, editing or post-production^(^ 10 ^)^. Planning is essential to ensuring the success of the subsequent steps, and this study presents the development and validation of a storyboard, which portrays (lines and images) exactly what is addressed in the video, in addition to guiding the filming and avoiding the need to re-editing^(^ 10 ^,^ 12 ^)^. The script was composed of four topics: definition, indication, procedure, and complications in order to address all the knowledge necessary to teach students and the population that will use this tool.

The Delphi Technique was used to validate the script. Even though it has seldom been used for this specific purpose, it has been used to validate instruments and manuals through expert consensus^(^ 11 ^)^. The experts were nursing professors from the Fundamental Nursing Program and professional nurses and the experience of judges on the topic was taken into account; both professionals had experience with this procedure (bed bath) in their professional routines^(^ 11 ^)^. The choice of 100% inter-judge agreement was intended to lend greater rigor to the planning phase, though a mean of 75% consensus is generally used^(^ 18 ^)^.

Six rounds were necessary to validate the script, a number larger than that reported in the literature (two to three rounds)^(^ 18 ^)^. We believe this result is due to the size of the script, which contained 18 pages, and the fact the experts did not communicate during the assessments, which might have hindered the clarification of potential divergences.

The main suggestions involving content were related to the procedure itself, such as keeping the bed rails up when positioning the patient in lateral decubitus; concomitantly cleaning the patient’s bed and furniture with a 70% alcohol; and removing the plastic protecting the venous access. These steps are essential to ensuring patient safety and reducing the risk of infection. Another suggestion was to initiate hygiene on the upper and lower limbs on the side as opposed to the professional who is bathing the patient. The reason is that another professional can simultaneously rinse and dry the patient. Even though this information is not emphasized in the literature, it was retained because it does not violate the basic principle of bed bathing, that of washing from the least to the most contaminated parts^(^ 19 ^)^. The information not to apply vigorous comforting massage on bone prominences and areas where pressure injuries are starting to appear was also included in the script, as guidelines concerning pressure injuries emphasize it. The objective of this information is to prevent and/or not worsen pressure injuries^(^ 20 ^)^. Another suggestion was to keep the call-light close to the patient after the bath, especially for the bedridden. One study emphasizes that not having devices to call the health staff is a risk factor for falls, which compromises the safety of patients^(^ 21 ^)^. Various experts suggested, especially the nursing professionals, that the modified bed bath technique used for unconscious patients and those unable to raise their hips for placement of the bedpan, was included. The experts suggested that after washing the patient’s feet, intimate hygiene be performed without placing the bedpan under the patient’s hips and then change the water, washcloth, and gloves in order to wash the patient’s back, buttocks and anus. This same sequence has been suggested when other devices are used for the bed bath, such as disposable washcloths; however, it is recommended to use each washcloth on one body part and dispose of it afterwards, minimizing cross infection^(^ 19 ^)^. Therefore, this modified technique was included, emphasizing changing the water, gloves, and washcloth after intimate hygiene.

The use of simulators prepares students for the real context of practice, helping them to develop practical skills and appropriate care delivery, along with patient privacy^(^ 1 ^)^. The nurse who played the role of a patient recorded scenes related to orientation provided by the health worker and scenes of the technique that did not involve body exposure. The use of professionals who experience the procedure in their practice substantiates the technique and facilitates recording^(^ 10 ^,^ 12 ^)^. The video’s final version, i.e., after editing, was 21 minutes and 21 seconds long. Studies suggest that a video should not last more than 10 to 20 minutes^(^ 10 ^)^, in order to avoid hindering comprehension of content on the part of viewers and also due to the difficulty disseminating the material on the internet^(^ 10 ^-^ 11 ^)^. In this case, however, parts of the video can be viewed separately, while the most extended topic is the description of the procedure, with 17 minutes and 24 seconds.

After the editing, the video was first watched by professors, who had participated in the script validation, so that they could identify potential gaps; a few adjustments were suggested and then implemented. One professor suggested changing the order of one of the scenes and keeping hand washing after organizing the room. Hand washing should take place before and after contact with patients and material that is close to patients (objects and surfaces close to the patient), in order to avoid the transmission of microorganisms from one patient to professionals or other patients^(^ 22 ^)^. Other suggestions included: raising the volume of the actors’ voices in order to make them more comprehensible, considering different environments would be used to transmit the video and also replacing the term pressure ulcer with pressure injury. The term “pressure ulcer” was recently changed to “pressure injury” because, according to the *National Pressure Ulcer Advisory Panel* - NPUAP, the expression pressure injury more accurately describes this type of injury, both in terms of intact and ulcerated skin^23^. After re-editing with the suggested changes, the video was watched by second-year nursing students. Sophomore students were chosen because they had seen this content a few months prior to the video presentation; thus they could be expected to understand it. The students considered the video to be comprehensible and did not suggest any adjustments.

Agreement between experts and the target population on the video concept, clarity, order of dialogue and language, is fundamental to ensure understanding of what is presented and renders the video more attractive. The use of an incorrect concept and/or terms difficult to understand may lead to the inappropriate performance of a procedure and, consequently, compromise patient safety^(^ 3 ^)^. Additionally, the use of confused and incomprehensible language can cause fatigue and disperse viewers, potentially leading to the incorrect performance of a procedure^(^ 3 ^)^.

This study contributes to the advancement of knowledge in Fundamental Nursing and the acquisition of practical skills by students and nurses and to improve teaching practices both in the classroom and in simulation centers. Additionally, it can contribute as a guide for the production of videos in different areas of knowledge.

The study’s limitation is related to the size of the script, which may have hindered the experts’ assessments.

## Conclusion

A video addressing bed bathing was developed and validated. The video was composed of four topics addressing the definition of bed bathing, the indications for this type of bath, steps to execute this procedure, and related complications. This study is expected to contribute to professional training and improvement of the knowledge and skills of nursing students.
